# Technological Advancements and Economics in Plant Production Systems: How to Retrofit?

**DOI:** 10.3389/fpls.2022.929672

**Published:** 2022-07-01

**Authors:** Daniel Dooyum Uyeh, Rammohan Mallipeddi, Tusan Park, Seungmin Woo, Yushin Ha

**Affiliations:** ^1^Department of Bio-Industrial Machinery Engineering, Kyungpook National University, Daegu, South Korea; ^2^Upland-Field Machinery Research Centre, Kyungpook National University, Daegu, South Korea; ^3^Smart Agriculture Innovation Center, Kyungpook National University, Daegu, South Korea; ^4^Department of Artificial Intelligence, School of Electronics Engineering, Kyungpook National University, Daegu, South Korea

**Keywords:** decision making, greenhouse, non-dominated sorting genetic algorithm, plant factory, return on investment, resilient food systems

## Abstract

Plant production systems such as plant factories and greenhouses can help promote resilience in food production. These systems could be used for plant protection and aid in controlling the micro- and macro- environments needed for optimal plant growth irrespective of natural disasters and changing climate conditions. However, to ensure optimal environmental controls and efficient production, several technologies such as sensors and robots have been developed and are at different stages of implementation. New and improved systems are continuously being investigated and developed with technological advances such as robotics, sensing, and artificial intelligence to mitigate hazards to humans working in these systems from poor ventilation and harsh weather while improving productivity. These technological advances necessitate frequent retrofits considering local contexts such as present and projected labor costs. The type of agricultural products also affects measures to be implemented to maximize returns on investment. Consequently, we formulated the retrofitting problem for plant production systems considering two objectives; minimizing the total cost for retrofitting and maximizing the yearly net profit. Additionally, we considered the following: (a) cost of new technologies; (b) present and projected cost for human labor and robotics; (c) size and service life of the plant production system; (d) productivity before and after retrofit, (e) interest on loans for retrofitting, (f) energy consumption before and after retrofit and, (g) replacement and maintenance cost of systems. We solved this problem using a multi-objective evolutionary algorithm that results in a set of compromised solutions and performed several simulations to demonstrate the applicability and robustness of the method. Results showed up to a 250% increase in annual net profits in an investigated case, indicating that the availability of all the possible retrofitting combinations would improve decision making. A user-friendly system was developed to provide all the feasible retrofitting combinations and total costs with the yearly return on investment in agricultural production systems in a single run.

## Introduction

Plant production systems offer numerous opportunities and benefits for growers, such as year-round cultivation, improved growing conditions for ornamental crops and vegetables, and control of micro- and macro- environments (Gerson and Weintraub, 2007; Van Straten et al., 2010; Nordey et al., 2017). These systems have been serving communities for decades. They have transformed from simple structures to grow vegetables in temperate regions during the cold winter months to advanced facilities currently used to grow in tropics, including deserts (Wittwer and Castilla, 1995; Gullino et al., 1999; Shamshiri et al., 2018).

The advancement in these protected cultivation structures is still ongoing, with the world incessantly requiring improvements to cater to the fast-growing populace demanding healthier food. Top on this list is the diminishing skilled farm labor, rapidly changing climate, and disasters such as the COVID 19 outbreak caused by the SARS-CoV-2 virus (Wang et al., 2020) that became widespread at the beginning of 2020, leading to difficulties in international travels for migrant workers (Lima et al., 2020). Most countries were forced to close their borders or place stringent entry procedures (Barua, 2020; Wells et al., 2020). This has led to various farm losses (Galanakis, 2020; Helm, 2020; Nicola et al., 2020; Sahoo and Rath, 2020). Autonomous growing has been under investigation to resolve labor accessibility and precision issues. Also, the environment in plant production systems is toxic to humans because of the poor ventilation and high temperature and humidity content. Advanced plant production systems are complex multi-input structures that come at a high cost (Stanghellini and Montero, 2010; Baeza et al., 2011; Reddy, 2016). This necessitates the proper implementation of new and/or existing technologies.

Plant production systems existed for centuries (42 BC–37 AC), but the major advancement occurred in the early 1950s and has continuously improved to the current phase (Jensen and Malter, 1995; Paul and Gwynn-Jones, 2003; Raviv and Antignus, 2004; Nordey et al., 2017). Most plant production systems, such as plant factories and greenhouses, were not designed to adopt the new technologies. Furthermore, building new structures also needs proper planning and implementation. This contrasts with the open field cultivation system that requires less planning. Plant production systems could be catastrophic if proper planning and implementation are neglected despite having positive returns. With the current evolution in technologies, the grower should have an appropriate decision-making system that considers investment capital, interest on loans, market opportunities, and profit, which are critical to sustainability.

Structural upgrades are often required for implementing new technologies. Retrofitting is usually adopted as the choice approach. This has been applied majorly to residential buildings to save energy and limit greenhouse gas emissions (Dixon et al., 2008), simultaneously considering several environmental and economic criteria (Antipova et al., 2014), comparing internal and external thermal insulation systems for residential buildings (Kolaitis et al., 2013), and several other retrofitted buildings with focus on energy saving (Xu et al., 2011, 2015; Xu and Chan, 2013; Wu et al., 2016; El-Darwish and Gomaa, 2017; Fan and Xia, 2017, 2018; Liu et al., 2018). In this scenario, the combination of retrofitting measures and strategies has proven to be complex and requires tradeoffs. In residential buildings, the measures adopted to retrofit the buildings for energy efficiency are categorized into the following groups: (a) measures to reduce load; (b) measures to control and monitor loads; (c) enveloping measures such as insulation and sealing roofs or ceilings; (d) alter energy consumption patterns of the occupants; and (e) adoption of renewable energy sources (Diakaki et al., 2008; Marszal et al., 2011; Ma et al., 2012; Malatji et al., 2013).

Retrofitting plant production systems to cover the progress in efficient growing technologies is much more complex than residential buildings that focus primarily on energy. The energy retrofitting benefits could be social, which has to do with enhancing the health and comfort of the occupants, reducing air emissions hurting the environment, and economic perspective in reduction of operation costs (Jafari et al., 2016). The dynamics in plant production systems are numerous, requiring multi-objective optimization approach, and the strategy to adopt and/or retrofit the existing system is much more challenging and delicate. These include (a) plant production systems are far more extensive than regular residential houses reaching 215 square feet (Robinson, 2018); (b) the advancement in technologies used in growing are occurring simultaneously in different aspects of protected cultivation and at a much faster rate than residential houses (Uyeh et al., 2019b; Raviteja and Supriya, 2020; Rayhana et al., 2020); (c) unlike in residential buildings where the primary concern is energy consumption for heating and cooling (Jafari and Valentin, 2017), plant production systems require energy for similar purposes in addition to other technological advancements such as autonomous growing that needs to be retrofitted (Bogue, 2020; Kurtser and Edan, 2020); and (d) wrong retrofitting strategy would not result in discomfort as in residential buildings but an irreversible loss of the plants accompanied with substantial economic losses. These make the retrofitting problem in protected cultivation non-deterministic polynomial-time hard (NP-hard) (Bagnall et al., 2001).

Multi-objective optimization requires maximizing or minimizing multiple objective functions that are constrained. These include analyzing design, selecting process designs or optimal products, tradeoffs, or applications where optimal solutions are needed with tradeoffs between two or more conflicting objectives. The conventional approaches for this type of optimization include the Pareto front, goal attainment, and minimax. In Pareto fronts, noninferior solutions are found. These are solutions in which an improvement in one objective requires a degradation in another.

On the other hand, goal attainment reduces the values of a linear or nonlinear vector function to attain the goal values given in a goal vector. The comparative significance of the goals is shown by applying a weight vector, and goal fulfillment problems may also be subject to linear and nonlinear constraints. Finally, minimax, minimizes the worst-case values of a set of multivariate functions, probably subject to linear and nonlinear constraints (MathWorks, 2021).

Multi-objective techniques are popular due to their capabilities in solving a wide range of real-world problems (Saini and Saha, 2021). For example, Fonseca and Fleming (1993) Multiple Objective Genetic Algorithm enables decision-makers to progressively articulate their preferences while learning about the problem under consideration. Srinivas and Deb’s (1994) Nondominated Sorting in Genetic Algorithms adopted Goldberg’s notion of nondominated sorting in genetic algorithms and a niche and speciation method to find multiple Pareto-optimal points simultaneously. Horn et al.’s (1993) Niched Pareto genetic algorithm, a multi-objective optimization algorithm, is adopted to find the Pareto optimal set. The previously discussed algorithms are some of the elitist multi-objective methods that non-dominated sorting genetic algorithm II (NSGA II) used in this study have been proven to be better (Deb et al., 2002). These methods are limited in their computational complexity (the number of objectives and population size), non-elitism approach; and the need for specifying a sharing parameter that alleviates all the above three difficulties.

In summary, Pareto optimality which is the backdrop on which NSGA II is built, has been reported to be the best approach to describe multi-objective optimization since there is no single global solution. It is often necessary to determine a set of points that all fit a predetermined definition for an optimum (Marler and Arora, 2004). NSGA II is undoubtedly the elitist method (Deb et al., 2000; Kannan et al., 2008; Li and Zhang, 2008; Yusoff et al., 2011). NSGA-II, a multi-objective evolutionary algorithm, improves the difficulties of using multi-objective optimization. These include the need to specify a sharing parameter, computational complexity, and a non-elitism approach. It possesses a selection operator that generates a mating pool by merging the parent and offspring populations and selecting the best N solutions (Deb et al., 2002).

Consequently, in this study, we formulated the protected cultivation retrofitting problem considering; (a) cost of retrofitting items such as sensors and robots; (b) cost of labor and cost-benefits obtainable from replacing human labor with robots; (c) size and service life of the plant production system; (d) impact of retrofitting on productivity and consequently profit; and (e) category of retrofitting to be implemented which delivers tradeoff solutions that represent the possible retrofits associated with expenditure and benefits. This problem was then solved using NSGA-II. Parameters such as present and projected cost of labor and agricultural products can be set to the user’s local context. Due to the conflicting nature of the objectives, NSGA-II can provide a tradeoff solution that can enable better decision-making when selecting retrofit measures. We demonstrated the applicability of this method by carrying out experimental simulations on different plant production system sizes.

## Retrofitting in Plant Production Systems

**FIGURE 1:**
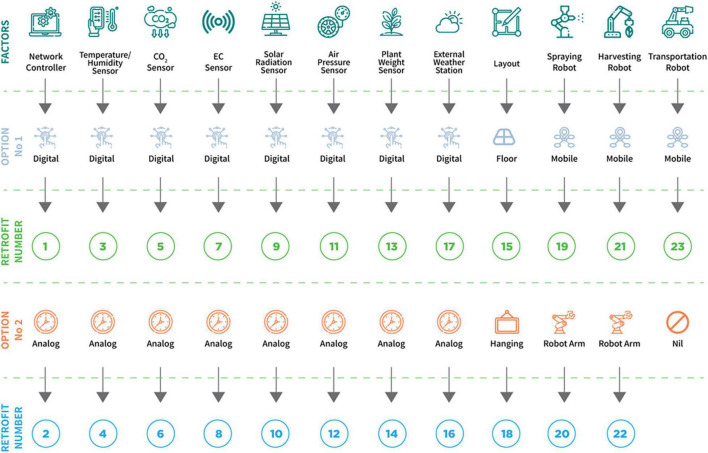
Retrofit factors and options used in the experimental simulation.

**FIGURE 2:**
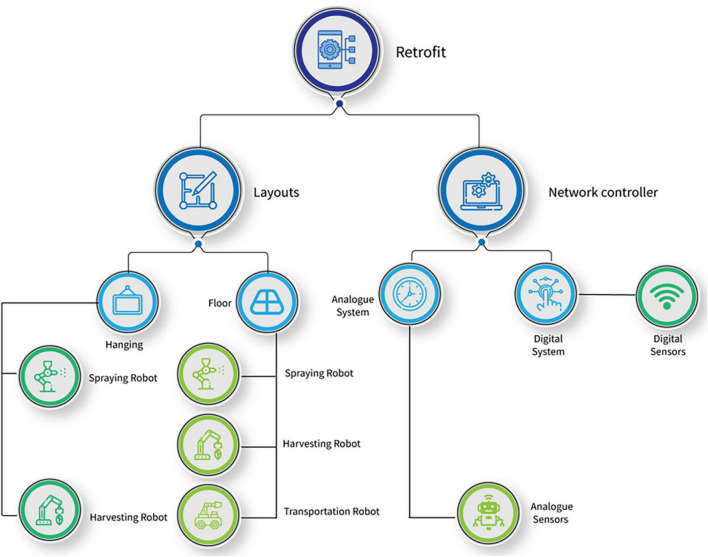
Tree showing constraints for implementing retrofits.

**FIGURE 3:**
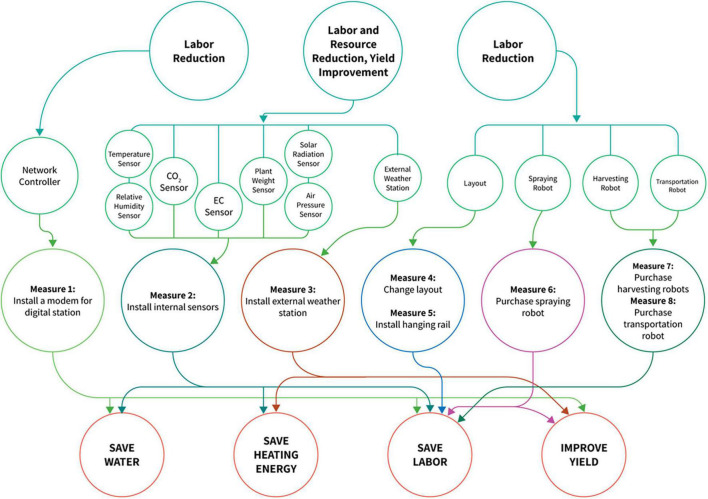
Retrofitting procedures, their interactions, and potential benefits.

Figure 1 shows the factors and options available for retrofitting a plant production system. In this study, a prospective retrofit is represented as “*RM*”. Furthermore, some options are limited by constraints, as shown in Figure 2. Two options are available in retrofitting the plant production system to include a network controller (Figure 2). If an analogous network controller is selected, all sensors to be selected must be analog. A similar procedure would occur if a digital network controller were selected and with the type of layout and robots, respectively. Retrofit number 23 (Transportation robot) was considered nil only in Option 2 because a transport robot is not required in this situation. Figure 3 shows the benefits derived from the combination of different retrofit measures.

The factors are represented with vector “*X*” as shown below:


X=[1, 2, 3, 4, 5, 6, 7, 8, 9, 10, 11, 12, 13,14, 15, 16, 17, 18, 19, 20, 21, 22, 23]

**Table:** 

*X*=	1	2	3	4	5	6	7	8	9	10	11	12
RM1=	[ℝ		ℝ		ℝ		ℝ		ℝ			
RM2=	[	ℝ		ℝ		ℝ		ℝ		ℝ		ℝ
.												
.												

**Table:** 

*X*=	13	14	15	16	17	18	19	20	21	22	23	
RM1=					ℝ		ℝ		ℝ		]	
RM2=						ℝ		ℝ		ℝ	]	
.												
.												

The prospective retrofit represented with vectors RM1, RM2,… above presents the feasible retrofit measures that could be implemented. The selected retrofit measure denoted with “ℝ” corresponds to the factor number (*X*) for a given feasible retrofit vector “RM”.

The retrofitting problem in a plant production system differs from conventional residential buildings. In this study and referring to the scenario in the Republic of Korea, the following variables were considered:

(a) Size of the plant production system.
(b) The service life of the plant production system (*m*).
(c) Cost of the items for retrofitting (*C*_*i*_).
(d) Cost of electricity per unit (*UCE*).
(e) Impact of retrofitting on electricity consumption (*ECC*).
(f) The initial estimated cost of energy consumption.
(g) Interest paid on loans for retrofitting items.
(h) The annual rate of increase in energy cost (*e*).
(i) Maintenance and replacement period for each retrofitted item (*t*_*RMi*_).
(j) Number of maintenance and replacements needed to be done during the service life of the system (*nRM*_*i*_).
(k) Production before and after retrofitting was done (*PBR*).
(l) Price per unit of production.
(m) Projections in the price of the product.
(n) Labor cost before and after retrofit.
(o) Projections in the cost of labor.
(p) Profit (*P*).

## Problem Formulation

### Expenditures in Retrofitting a Plant Production System

#### Initial Cost of Investment

To calculate the initial cost of investment (*ICI*) to retrofit in a plant production system, the cost of purchasing sensors (digital or analog) for precision and improved decision making, retrofitting the navigation system for the robots (mobile rail or hanging system), and purchase cost of robots were considered and computed in Eq. 1.


(1) $$I\,C\,I=\sum_{i=1}^{n}C_{i}\,y_{i}$$

Where *C*_*i*_ is the cost of implementing the *i*th retrofitting measure, which is the cost of the items for retrofitting, and *y*_*i*_ is an indication variable demonstrating if the *i*th retrofitting measure is selected in the automation strategy. Furthermore, *n* is the total number of potential retrofitting measures.

#### Energy Consumption Cost

To compute the current energy consumption cost from retrofitting (*ECC*) the protected cultivation system, Eqs 2, 3 were used (Fan and Xia, 2018).


(2) $$E\,C\,C=Y\,E\,C\times \left[\frac{{\left(1+\left(\frac{I\,r-e}{1+e}\right)\right)}^{m}-1}{\left(\frac{I\,r-e}{1+e}\right)\times {\left(1+\left(\frac{I\,r-e}{1+e}\right)\right)}^{m}}\right]$$

Where *YEC* is the estimated yearly energy consumption cost of the plant production system in the first year, *Ir* is the interest rate, *e* is the annual rate of energy cost increase (a rate of 5% was considered), and *m* is the service life of the plant production system.

The yearly energy consumption of the plant production system in the first year can be calculated as the sum of the estimated electricity per year as follow:


(3) $$A\,E\,C=\left(\sum_{i=1}^{n}E\,C_{i}\right)\times U\,C\,E$$

Where *AEC* is the annual energy consumption of the plant production system before implementing the energy retrofit, *EC* is the energy consumption of the items in retrofitting the plant production system, and *UCE* is the unit cost of electricity.

#### Replacement and Maintenance Cost

To estimate the replacement and maintenance cost because of retrofitting the plant production system, the number of replacements during the service life of the system is calculated using Eqs 4, 5 (Fan and Xia, 2018):


(4) $$n\,R\,M_{i}=R\,o\,u\,n\,d\,\_\,D\,o\,w\,n\,\left[\frac{\left(t\right)}{t_{R\,M_{i}}}\right]$$

Where *nRM*_*i*_ is the number of times replacements and maintenance are required for the *i*th measure during the service life of the plant production system, and *t*_*RMi*_ is the replacement and maintenance period for the *i*th measure.

Furthermore, to compute the current replacement and maintenance cost from the retrofits, the equation below was used.


(5) $$E\,C\,C_{R\,c}=\sum_{i=1}^{n}\left[\left(\sum_{j=1}^{n\,R\,M_{i}}\frac{E_{M\,R\,i}}{{\left(1+p\right)}^{j}\times t_{R\,M_{i}}}\right)\right]\times x_{i}$$

Where *E*_*MRi*_ is the expenditure estimated from replacement and maintenance to implement the *i*th activity after its replacement and maintenance period.

#### Total Expenditure in Retrofitting a Plant Production System

The total expenditure is computed using Eq. 6:


(6) $$E\,x\,p\,e\,n\,d\,i\,t\,u\,r\,e=C\,R+E\,C\,C+E\,C\,C_{R\,C}$$

Where *CR* is the cost of the systems used in the retrofits, *ECC* is the current energy consumption cost from retrofitting the system and *ECC*_*RC*_ is the replacement and maintenance cost for the retrofitted items.

### The Net Profit Derived From Retrofitting a Plant Production System

The profit gotten from the retrofit is assumed from two perspectives in this study. These were computed using Eqs 7–10.

#### Net Profit From Improved Productivity

This was calculated as follows:


(7) $$P\,r\,o\,f\,i\,t_{P}=Y\,E\,C-P\times \left[\frac{{\left(I+\left(\frac{I\,r-e}{1+e}\right)\right)}^{m}-1}{\left(\frac{I\,r-e}{1+e}\right)\times {\left(1+\left(\frac{I\,r-e}{1+e}\right)\right)}^{m}}\right]$$

Where *YEC* is the estimated yearly energy consumption cost of the plant production system in the first year, *P* is profit from the retrofit, *Ir* is the interest rate, *e* is the annual rate of energy cost increase (a rate of 5%), and *m* is the service life of the plant production system.


(8) $$Y\,E\,C_{P}=E\,L_{P}\times U\,E\,L_{P}$$

Where *YEC*_*P*_ is the estimated yearly energy cost of production in the plant production system for the first year, *EL*_*P*_ is estimated annual production in the first year due to retrofit, and *UEL*_*P*_ is the price per unit productivity.

#### Net Profit From Savings in the Cost of Labor


(9) $$P\,r\,o\,f\,i\,t_{L}=Y\,E\,C-L\times \left[\frac{{\left(I+\left(\frac{I\,r-e}{1+e}\right)\right)}^{m}-1}{\left(\frac{I\,r-e}{1+e}\right)\times {\left(1+\left(\frac{I\,r-e}{1+e}\right)\right)}^{m}}\right]$$

Where *YEC* is the estimated yearly energy consumption cost of the plant production system in the first year, *L* is the Labor cost, *Ir* is the interest rate, *e* is the annual rate of energy cost increase (a rate of 5%), and *m* is the service life of the plant production system.

Consequently, the total net profit, which is the increased income from added productivity due to the new items used in retrofitting the plant production system and the money saved from labor spending because of the new systems that were retrofitted and replaced labor cost was calculated as:


(10) $$P\,r\,o\,f\,i\,t=P\,r\,o\,f\,i\,t_{P}+P\,r\,o\,f\,i\,t_{L}$$

### Optimization Model

The optimization problem was formulated with two objectives: to minimize the total expenditure to retrofit for the lifespan of the plant production system (Eq. 11) while maximizing the yearly net profit derived from retrofitting the system (Eq. 12). This is shown below as objectives 1 and 2.

#### Objective 1: Expenditure for Retrofitting a Plant Production System


(11) $$M\,i\,n\,i\,m\,i\,z\,e\,E\,x\,p\,e\,n\,d\,i\,t\,u\,r\,e=C\,R+E\,C\,C+E\,C\,C_{R\,C}$$

Where *CR* is the cost of the systems used in the retrofits, *ECC* is the energy consumption cost because of retrofitting new systems and *ECC*_*RC*_ is the replacement and maintenance cost for the retrofitted items.

#### Objective 2: Net Profit From Retrofitting a Plant Production System


(12) $$M\,a\,x\,i\,m\,i\,z\,e\,P\,r\,o\,f\,i\,t=P\,r\,o\,f\,i\,t_{P}+P\,r\,o\,f\,i\,t_{L}$$

The profit from the retrofit is the summation of the increased income from added productivity due to the new items used in retrofitting the plant production system and the money saved from labor spending because of the new systems that were retrofitted and replaced labor costs.

#### Constraints in Carrying Out Retrofits

In addition to the two objectives, the following constraints were implemented in this study, as shown earlier in Figure 2. The problem formulation could be tuned to incorporate other constraints depending on the system.

##### Selection of Sensors and Network Controller for Retrofitting

Since digital network controllers are meant to transmit data remotely, the type of sensors that could synchronize with it must have certain features. We formulated a constraint that only sensors with this capacity should be picked if a digital network controller is selected. This was also extended to the on-site network controller (Eq. 13).


(13) x(2(i-1)x(2(i-1)+2)=0

Given that, *i* = 1……..11 (retrofitting number).

##### Selection of Layouts for Retrofitting

With the current advances in plant production systems, two types of robotic navigation systems have been studied. These are mobile robots that navigate on the floor of the plant production system (Uyeh et al., 2019b) and hanging types of robots suspended above the plants and hung to the roof of the plant production system. In this constraint, the problem is formulated that if the hanging type of layout is picked for retrofit, then the selected harvesting and spraying robots should be robot arms, and no transportation robot should be chosen. If otherwise, then all types of robots could be selected (Eqs 14, 15).


(14) $$\text{max}\,\left(x\,\left[19\right],x\,\left[21\right],x\,\left[23\right]\right)\times x\,{\left[17\right]}^{1}=0$$

(15) $$\text{max}\,\left(x\,\left[20\right],x\,\left[22\right]\right)\times x\,{\left[18\right]}^{1}=0$$

Where *x* is the number of item for retrofit.

### The Search Algorithm Used in This Study

**FIGURE 4:**
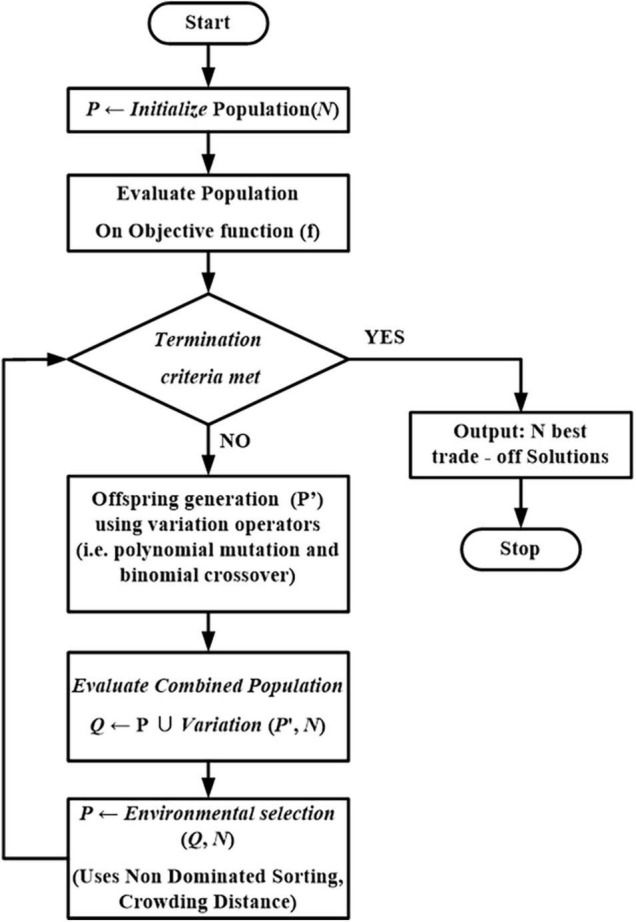
Flowchart of non-dominated sorting genetic algorithm.

The two objectives considered in the study – (a) minimization of investment cost and (b) maximizing the profit, are conflicting. In other words, minimizing investment costs results in lesser profits while maximization of profits demands more investments. Therefore, the optimization of multi-objective optimization problems does not provide a single optimal solution but a set of tradeoff solutions referred to as Pareto-optimal solutions. Population-based evolutionary algorithms are considered to solve the multi-objective problem due to their effectiveness and ability to provide the entire tradeoff solutions in a single run. Specifically, NSGA-II (Deb et al., 2002) is a more popular multi-objective evolutionary algorithm and has been widely adopted for real-world optimization problems. The general flowchart of NSGA-II is shown in Figure 4. NSGA-II starts with a randomly generated population of size (*N*), whose objective values are evaluated. The initialized population evolves over the generations through variation operators such as mutation, crossover, and environmental selection. The variation operators aim to produce effective solutions (referred to as offspring members) by using the information present in the solutions of the current population (referred to as the parent population).

On the other hand, environmental selection aims to select effective solutions from the combination of parent and offspring populations (P). In other words, environmental choice drives the entire population toward convergence to the Pareto-optimal solutions. The process of producing offspring members and environmental selection is repeated until the termination criteria are met. The variation operators considered in the current study are polynomial mutation and binomial crossover. Multi-objective optimization aims to obtain a set of converged well-spread diverse Pareto-optimal solutions. Thus, in NSGA-II, the environmental selection is made using non-dominated sorting followed by crowding distance, which is supposed to provide convergence and diversity. Non-dominated sorting and crowding distance are used in NSGA II to obtain the Pareto dominance of final tradeoff solutions (Uyeh et al., 2019a). The parameters of the optimization algorithm were set as follows:

Maximum number of generations (termination criteria): 500.

Population size (N): 500.

Crossover: Simulated binary crossover.

Constraint bond: 0–20.

Distribution indices for mutation (nm): 20.

Distribution indices for crossover (nc): 20.

Probability of crossover (Pc): 1.0.

Probability of mutation (Pm): 1/10.

Mutation: Polynomial mutation.

The average run time for the proposed algorithm was 180 s. The simulations were done on a 3.59 GHz AMD Ryzen 5 3500X 6-Core processor, 16 GB random access memory, and 256 GB solid-state drive with Windows 10 operating system in MATLAB (Matlab and Simulink, 2012). We conducted several simulations using guidelines from a previous manuscript (Deb et al., 2002) that proposed the algorithm and our experience working with this algorithm (Uyeh et al., 2018, 2019a,b). We finetuned and gradually increased the generations (iteration) until we got no further improvements. The number of generations that converged served as a termination value.

### Experimental Design and Data Used in the Simulation

**FIGURE 5:**
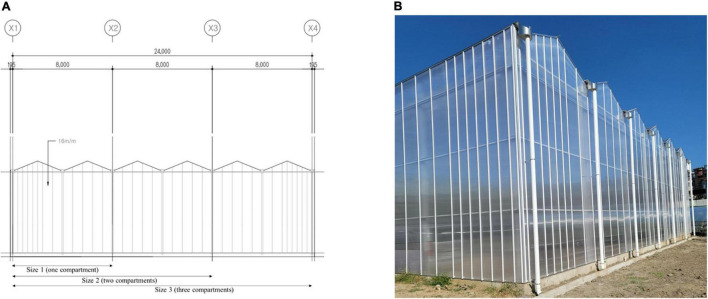
Schematic of the experimental plant production system with one to three compartments (X1–X2: Size 1, X2–X3: Size 2, X3–X4: Size 3) **(A)**, and external view of the system **(B)**.

To evaluate the robustness of the proposed method, two sub-factors of the investigated factors were considered (Table 1) with three Cases and five sizes of a plant production system. The plant production system used for this study had five compartments of similar sizes. Size one represented one compartment, size two represented two compartments, and up to size five represented all compartments. The schematic is shown in Figure 5. Usually, growers have their systems divided into compartments of similar sizes for different reasons, such as ease of management. Protected cultivation systems are typically single large structures divided into smaller simple compartments. Depending on the local situation and resources of the grower, the system could be divided into various compartments (Research Wua, 2021). For example, The Radix Serre Plant production system in the Netherlands has 9,000 m^2^ glass and comprises over 100 compartments (Research Wua, 2021). Each is considered and treated as an individual system. This study selected a plant production system with one to five compartments. Depending on the factor (type of equipment) and the number of compartments, the relationship between the variables at different sizes (compartments) would be linear as the compartments would require the same number of equipment such as the sensors (temperature and humidity). Since the compartments in the protected cultivation system have similar sizes, cost, impact on electricity consumption, production, and labor cost had a linear relationship. When it comes to the cost of maintenance and replacement, it can be linear in some situations and not linear in others, as there are ranges that these are priced. For example, only a single network controller is required in a plant production system irrespective of the number of compartments; the cost of maintenance and replacement of the network controller would not be linear compared to temperature and relative humidity sensors. The number of sensors and other retrofitting measures in one compartment (Size 1) of the plant production system was selected based on Korean industrial standards (UBN, 2021; Table 2). A compartment of the system had a height of 6,700 mm, a width of 8,000 mm, and a length of 16,700 mm. This formed the basis for selecting the number of retrofitting measures required for the other system sizes (Sizes 2–5).

**TABLE 1:** Cases used in the experimental simulations.

Case	Production before retrofits (kg)	Labor cost (USD)
1	1,000	1,000
2	2,000	1,000
3	1,000	2,000

**TABLE 2:** Data used in the experimental simulation.

Factor	Category	Size 1					Size 2					Size 3					Size 4					Size 5				
		Cost (USD)	IOEC	CMR	Ip	IL	Cost (USD)	IOEC	CMR	Ip	IL	Cost (USD)	IOEC	CMR	Ip	IL	Cost (USD)	IOEC	CMR	Ip	IL	Cost (USD)	IOEC	CMR	Ip	IL
Network controller	A	1800	200	100	2	0.9	3600	200	100	4	1.8	5400	210	100	6	2.7	7200	220	100	8	3.6	9000	230	100	10	4.5
	AN	1260	140	60	1	0.5	2520	280	90	2	1	3780	420	120	3	1.5	5040	560	120	4	2	6300	700	150	5	2.5
Temperature/humidity	A	300	20	100	2	0.001	600	40	150	4	0.002	900	60	200	6	0.003	1200	80	200	8	0.004	1500	100	250	10	0.005
	AN	210	14	60	1	0.001	420	28	90	2	0.002	630	42	120	3	0.003	840	56	120	4	0.004	1050	70	150	5	0.005
CO_2_	A	345	20	100	2	0.001	690	40	150	4	0.002	1035	60	200	6	0.003	1380	80	200	8	0.004	1725	100	250	10	0.005
	AN	241.5	14	60	1	0.001	483	28	90	2	0.002	724.5	42	120	3	0.003	966	56	120	4	0.004	1207.5	70	150	5	0.005
EC	A	320	20	100	1	0.001	640	40	150	2	0.002	960	60	200	3	0.003	1280	80	200	4	0.004	1600	100	250	5	0.005
	AN	224	14	60	0.5	0.001	448	28	90	1	0.002	672	42	120	1.5	0.003	896	56	120	2	0.004	1120	70	150	2.5	0.005
Solar radiation	A	420	20	100	2	0.001	840	40	150	4	0.002	1260	60	200	6	0.003	1680	80	200	8	0.004	2100	100	250	10	0.005
	AN	294	14	60	1	0.001	588	28	90	2	0.002	882	42	120	3	0.003	1176	56	120	4	0.004	1470	70	150	5	0.005
Air pressure	A	62	20	100	1	0.001	124	40	150	2	0.002	186	60	200	3	0.003	248	80	200	4	0.004	310	100	250	5	0.005
	AN	43.4	14	60	0.5	0.001	86.8	28	90	1	0.002	130.2	42	120	1.5	0.003	173.6	56	120	2	0.004	217	70	150	2.5	0.005
Plant weight	A	50	20	100	1	0.001	100	40	150	2	0.002	150	60	200	3	0.003	200	80	200	4	0.004	250	100	250	5	0.005
	AN	35	14	60	0.5	0.001	70	28	90	1	0.002	105	42	120	1.5	0.003	140	56	120	2	0.004	175	70	150	2.5	0.005
External weather station	A	2000	200	100	1	0.001	4000	400	150	2	0.002	6000	600	200	3	0.003	8000	800	200	4	0.004	10000	1000	250	5	0.005
	AN	1400	140	60	0.5	0.001	2800	280	90	1	0.002	4200	420	120	1.5	0.003	5600	560	120	2	0.004	7000	700	150	2.5	0.005
Layout	F	50000	0	10000	0	0.5	100000	0	15000	0	1	150000	0	20000	0	1.5	200000	0	20000	0	2	250000	0	25000	0	2.5
	H	25000	0	600	0	0.1	50000	0	900	0	0.2	75000	0	1200	0	0.3	100000	0	1200	0	0.4	125000	0	1500	0	0.5
Spraying robot	M	30000	1200	6000	1	0.95	60000	2400	9000	2	1.9	90000	3600	12000	3	2.85	120000	4800	12000	4	3.8	150000	6000	15000	5	4.75
	R	10000	840	3600	0.5	0.95	20000	1680	5400	1	1.9	30000	2520	7200	1.5	2.85	40000	3360	7200	2	3.8	50000	4200	9000	2.5	4.75
Harvesting robot	M	30000	1200	6000	0.01	0.95	60000	2400	9000	0.02	1.9	90000	3600	12000	0.03	2.85	120000	4800	12000	0.04	3.8	150000	6000	15000	0.05	4.75
	R	10000	840	3600	0.005	0.95	20000	1680	5400	0.01	1.9	30000	2520	7200	0.015	2.85	40000	3360	7200	0.02	3.8	50000	4200	9000	0.025	4.75
Transportation robot	M	15000	1200	3000	0.01	0.95	30000	2400	4500	0.02	1.9	45000	3600	6000	0.03	2.85	60000	4800	6000	0.04	3.8	75000	6000	7500	0.05	4.75

*A, digital; AN, analogue; F, floor; H, hanging; M, mobile; R, rail; IOEC, impact on electricity consumption; CMR, cost of maintenance and replacement; IP, impact on production; IL, impact on labor. Source: Ubn (2021).*

The yield data (Table 1) used was guided by visits to plant production system growers in the Republic of Korea to validate the optimization model. Strawberry yields are dependent on the environmental conditions, systems, techniques of production, and type of plant production system, which includes plant factories and greenhouses, as also reported by Kubota (2015).

### Computation of Impact of Automation on Labor

Data were acquired using structured questionnaires from growers adopting plant production systems to demonstrate the importance of retrofitting robotics in a plant production system. The human category was divided into three groups based on their expertise. Finally, we considered a real-world scenario of the first-of-its-kind strawberry harvesting robot (Table 3) as a comparison.

**TABLE 3:** Comparison between humans and robots in strawberry harvesting.

Factors	Category (s)			
	Robot	Human		
		Beginner	Average	Experienced
Platform movement/Movement to fruit location	4.7	2	2	2
Fruit localization/identification	3.7	10	10	10
Obstacle localization	3.0	1	1	1
Visual servoing/harvesting decision making	4.0	10	7.5	5
Detach fruit	2.2	2	1.5	1
Put fruit in the container	7.8	5	4	3
Working time (per h/day)	20	4	6	8
Success rate (%)	95	Uncertain	Uncertain	Uncertain
Cost of purchase (USD)	110,000	Not applicable	Not applicable	Not applicable
Lifespan (years)	7	Not applicable	Not applicable	Not applicable
Salvage cost (Purchase cost/lifespan), USD	15,714	Not applicable	Not applicable	Not applicable

The factors (Sweeper, 2020) given in Table 3 were considered in deciding the impact of operating a robot on labor cost and yield.

Additionally, there are numerous benefits of using robotics in a plant production system that is near impossible to quantify in terms of monetary benefits but rather impact. These include:

(i) Safety of products.

(ii) Availability of skilled workers.

(iii) Incessant increase in wages of skilled workers as seen in the context of the Republic of Korea and other OECD countries.

Also, the data for the sensors were collected from the UBN sensors company (UBN, 2021) and used in the simulation.

Overall, the developed system provides the user with the possibilities of specifying their local context (Size and service life of the plant production system, cost of the items for retrofitting, cost of electricity per unit and impact of retrofitting on electricity consumption, interest paid on loans for retrofitting items, the annual rate of increase in energy cost, maintenance, and replacement period for each retrofitted item, projected production before and after retrofitting, projections in the price of the product, labor cost before and after retrofit, projections in the cost of labor and profit).

## Simulation Results and Discussion

### Measures, Cost of Expenditure, and Profit for Retrofitting Case 1 Plant Production Systems

**FIGURE 6:**
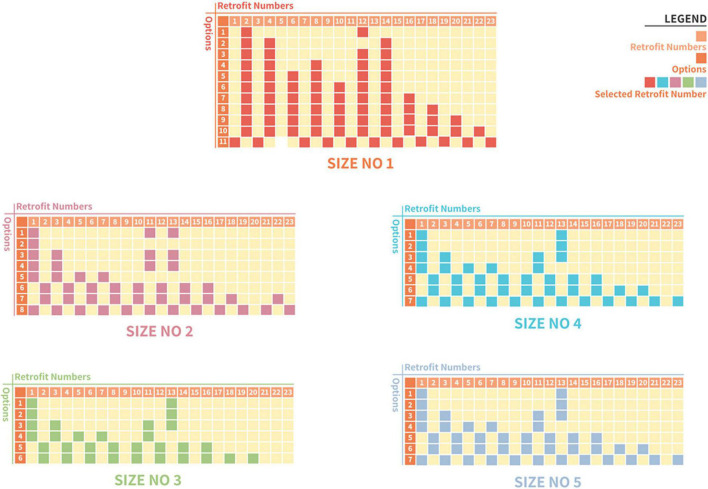
Generated retrofitting measures from the available factors for Case 1.

**FIGURE 7:**
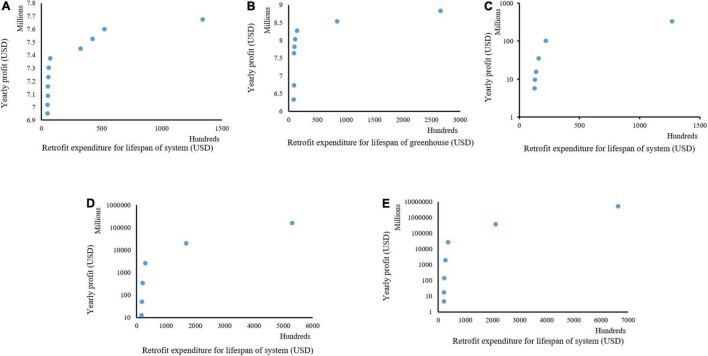
Different combinations of cost of retrofitting and yearly profit derived at different sizes of a plant production system for Case 1; size 1 **(A)**, size 2 **(B)**, size 3 **(C)**, size 4 **(D)**, and size 5 **(E).**

The simulation results show feasible combinations at different sizes for Case 1, represented with different colors for the selected measures (Figure 6). Each combination shows the total expenditure required to carry out the retrofit for the lifespan of the plant production system and the projected net profit per year (Figure 7). The feasible retrofits and tradeoff total expenditure versus the net profit per year are presented for all sizes. In Case 1, in size 1 of the retrofitting combinations, multiple feasible combinations were obtained compared to sizes 2–5 (Figure 6). However, the few possible retrofit combinations in sizes 2–5 show more return on investments (ROI) than the multiple feasible combinations in size 1. This demonstrates that despite the grower with size 1 having numerous possible combinations, the size of a system is more critical for profitability (Figure 7). The results also indicate that introducing new technologies might not necessarily mean a return on investment in an optimum way without analyzing all possible factors, such as current and projected labor costs and electricity consumption. The results in Figure 6 also show that despite a similar amount of money being spent to carry out the retrofit at a point across the different sizes of the plant production system, the net profit increased with the size of the system.

Further analyses of the return on investment in the size 1 (Figure 7A) in Case 1 showed a similar cost. Subsequently, retrofit combinations were available to be implemented that significantly increased the net profits. For example, there was a 4.03% increase in yearly net profit between two retrofit combinations with an investment cost of 4,990 and 5,700 USD (Figure 7A). An increase of 700 USD investment would result in about 280,000 USD or 4.03% in yearly net profit in this situation. In these combinations, the combination at the cost of 4,990 USD had selected retrofit measures 2 and 12 (Figure 6), which are analog network controller and temperature/humidity sensors (Figure 1). However, in the combination of 5,700 USD, the selected retrofit measures were 2, 4, 6, 8, 12, and 14 (Figure 6). This combination picked additional measures in addition to the two chosen at the cost of 4,990 USD. These were sensors for CO_2_, solar radiation, air pressure, and plant weight (Figure 6). Both combinations picked only analog measures. The sensors picked at the cost of 5,700 USD facilitated improved decision-making, thus increasing yearly productivity and extension profit.

Further analysis showed that all cost combinations except one selected the analog category instead of the digital. However, despite the investment cost of about 133,984 USD compared to the closest cost combination of about 52,394 USD which is less than half, the return on investments is approximately 7,675,358 USD and 7,599,365 USD, respectively (Figure 7A). This was a 1% increase compared to the 155% increase in investment cost. This analysis shows the importance of this system and the need to consider various factors when carrying out retrofits.

### Measures, Cost of Expenditure, and Profit for Retrofitting Case 2 Plant Production Systems

**FIGURE 8:**
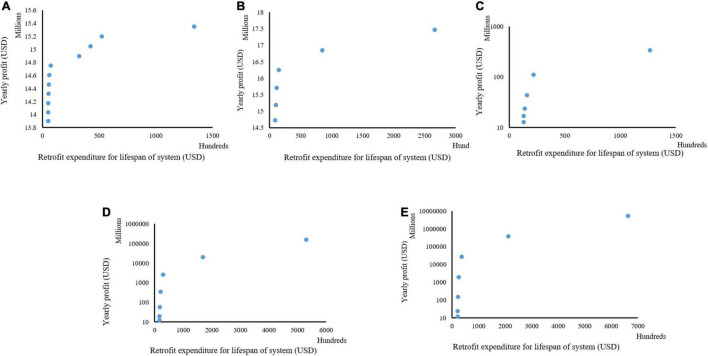
Different combinations of cost of retrofitting and yearly profit derived at different sizes of a plant production system for Case 2; size 1 **(A)**, size 2 **(B)**, size 3 **(C)**, size 4 **(D)**, and size 5 **(E).**

In Case 2 retrofitting measures for a plant production system (Figure 8), an increase in productivity at a similar labor cost in Case 1 was investigated. These analyses were done to ascertain the impact of production on retrofit. Despite the cost of investment was similar, there was a significant increase in the return on investment when the productivity was doubled. This was around a 100% increase in the return on investment in size 1 of the system (Figure 8A). However, as the size of the system increased (Figures 8B–E), despite the similarity in the investment cost for retrofitting between Case 1 and 2, and the doubling of the productivity, a different trend was seen with the return on investment of around 132% for sizes 2 (Figures 7B, 8B), 121% for sizes 3 (Figures 7C, 8C), 136% for sizes 4 (Figures 7C, 8D), and 155% for size 5 (Figure 8E). This demonstrates that the size of the system and productivity are essential factors to consider in retrofitting. A similar trend in combinations of retrofit measures to be implemented was seen between Case 1 and Case 2.

### Measures, Cost of Expenditure, and Profit for Retrofitting Case 3 Plant Production Systems

**FIGURE 9:**
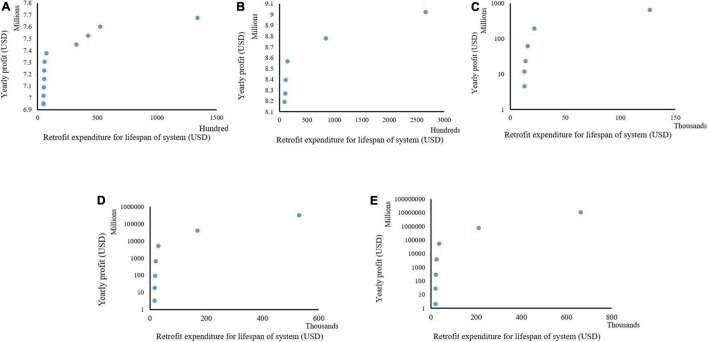
Different combinations of cost of retrofitting and yearly profit derived at different sizes of a plant production system for Case 3; size 1 **(A)**, size 2 **(B)**, size 3 **(C)**, size 4 **(D)**, and size 5 **(E).**

Case 3 was designed to investigate the impact of labor cost on the retrofit measures, cost of investment, and return on investment. This case was investigated because of the similarities in productivity that are sometimes found in plant production systems from the optimal control of the micro- and macro- environments but the difference in the local contexts with labor cost because of the disparity in standard of living and development. In this Case, the productivity was kept like in Case 1, but the labor cost was doubled in Case 3. Size 1 (Figure 9A) showed no difference between Case 1 and Case 3 at comparable investment costs, even though the labor cost in Case 3 doubled that in Case 1. However, in the size 2 (Figure 9B) of Case 3, a different trend was seen compared to Case 1. The results show that the least amount of money for retrofit (around 9,000 USD) had a better return on investment than the most expensive combination (about 26,000 USD) for retrofits in Cases 1 and 3. These were a 29% increase in return on investment in Case 1 compared to Case 3 at the least combination of retrofit factors and around a 2% increase in return on investment in Case 1 compared to Case 3 for the maximum combination of retrofit factors. This was even more with the comparisons in investment cost and the cost of labor in Case 3 being a 100% increase from Case 1. To validate our method, we analyzed the components selected in both situations (least and highest cost of investment). Only two retrofit measures were selected at the least cost of investment: a digital network controller and an air pressure sensor for Case 1. In Case 2, only the digital network controller was selected. The selected retrofit measures in both Case 1 and Case 3 have minimal impact on productivity and cost of labor. This verifies the increase in return on investment of around 29% in Case 1, size 2 from that of Case 3. The increase was because of the savings from labor costs in Case 1. However, with the maximum investment cost, all the automated measures were selected in Case 1 and Case 3. With this, the labor cost did not significantly impact the return on investment, thus leading to only a 2% increase in return on investment of Case 1 compared to Case 3.

#### Impact of Labor Cost and Productivity on Return in Investment in Retrofitting

Figures 7–9 shows the combination of the total expenditure required to carry out the retrofit for the lifespan of the plant production system and the projected net profit per year for three investigated cases. These cases varied in the production quantity before retrofits and local labor costs. These were done to explore what would impact the retrofitting expenditure and profits as different yields are gotten across different systems depending on the cultivated variety and other inputs. The labor cost also varies with the local situation as systems closer to the urban centers would have more expenditure on labor costs than those located farther from the cities. Analyses of the results showed that despite having similar spending for the three investigated scenarios of labor cost and yields, the total maximum yearly profit was similar for Cases 1 and 3 but was double for Case 2 from what was recorded in Cases 1 and 3 for size 1. A similar scenario was recorded in Size 2. In sizes 3–5, a similar maximum profit was recorded in all the investigated cases. This points out that despite the labor cost being double in Case 3, the similar productivity in Cases 1 and 3 would result in similar profits at a smaller production capacity, but this would change as the size of the production system increases. Also, our analyses show that at a smaller production capacity, the grower needs to pay attention to the best variety for productivity when retrofitting. This becomes less important when the size of the system increases. These analyses point to the importance of this decision-making tool when deciding to retrofit.

## Conclusion

A user-friendly system to generate all the feasible tradeoff retrofit combinations for agricultural production systems such as plant factories and greenhouses was developed in this study. Cost of new technologies, interest on loans for retrofitting, size and service life of the production system, the present and projected cost for human labor and robotics, productivity, energy consumption, and replacement and maintenance costs were considered in the developed system. The presentation of tradeoff solutions of possible retrofit combinations, total expenditure, and net profit per year that is made possible by the developed system would improve decision-making. For example, an investigated case showed an increase of up to 250% in net profits. We propose a multi-objective retrofitting method for agricultural production systems to minimize the total cost of investment and maximize the yearly net profit.

## Data Availability Statement

The original contributions presented in this study are included in the article/Supplementary Material, further inquiries can be directed to the corresponding author.

## Author Contributions

DU: conceptualization, methodology, software, investigation, formal analysis, data curation, visualization, and writing – original draft. RM: methodology, investigation, software, data curation, visualization, and writing – review and editing. TP and YH: validation, resources, writing – review and editing, supervision, and funding acquisition. SW: validation, visualization, funding acquisition, and project administration. All authors contributed to the article and approved the submitted version.

## Conflict of Interest

The authors declare that the research was conducted in the absence of any commercial or financial relationships that could be construed as a potential conflict of interest.

## Publisher’s Note

All claims expressed in this article are solely those of the authors and do not necessarily represent those of their affiliated organizations, or those of the publisher, the editors and the reviewers. Any product that may be evaluated in this article, or claim that may be made by its manufacturer, is not guaranteed or endorsed by the publisher.
