# Supplementary figures and images for: Technological Advancements and Economics in Plant Production Systems: How to Retrofit?
Source: Front Plant Sci. 2022 Jul 1;13:929672. doi: 10.3389/fpls.2022.929672 (PMC9289745; doi:10.3389/fpls.2022.929672)

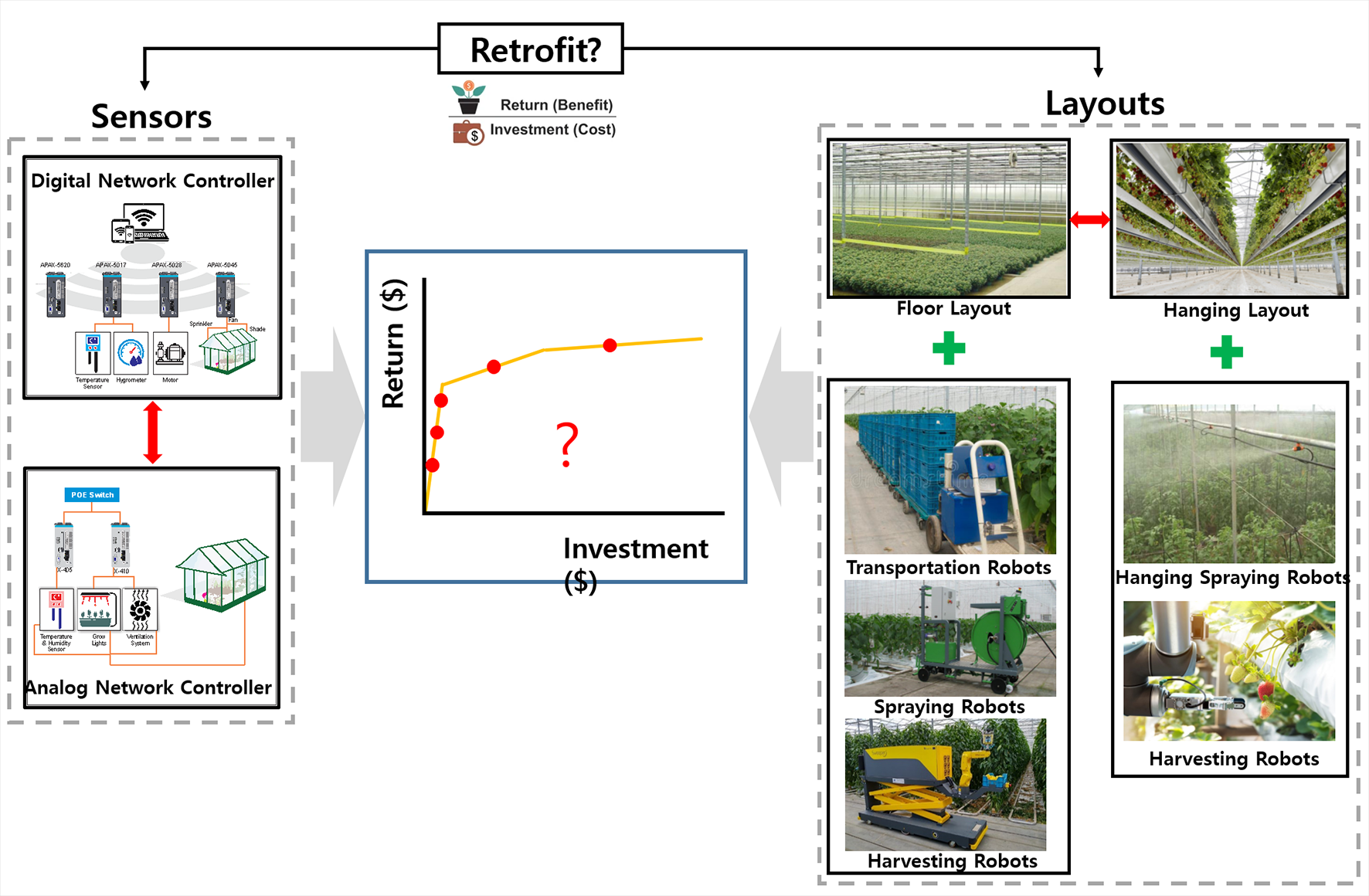

Supplement: Supplementary file 1 [file Image_1.TIF]
